# Little Things

**Published:** 1866-05

**Authors:** J. A. Robinson

**Affiliations:** Jackson, Mich.


### LITTLE THINGS.

BY J. A. ROBINSON, D. D. S., JACKSON, MICH.

Read before the Michigan Dental Association.

The common excuse among those attending this convention for not presenting any written document for our consideration is "that they cannot write in a manner to instruct or edify their fellows; or, that they cannot find anything *new* to say upon any of the subjects under discussion."

In commencing a great Epic Poem, a distinguished writer has said:

I want a hero, an uncommon want,
Since every day and month sends forth a new one;
Till after cloying the Gazettes with cant,
The age discovers he is not the true one.

In casting about for a subject, I have not sought for a hero, but have chosen the smallest subject, and the smallest things; and if they are treated in a small way, you may say it is owing to the subject, or the small man, who treated it according to his capacity.

It is the atoms that make this great world; and the valleys and fertile fields are made from what the great mountains have cast away in the smallest particles, or the unseen deposits that have floated silently down the rivers. It is the invisibles that cannot be seen or felt, that help to grow the corn, and the hay, and every vegetable production that covers this great and beautiful earth. So it is the insensible perspiration that is quietly and silently passing out of the open doors of our physical organism that preserves our life and health. It is the little virtues of men and women that make them the great men and women of the world; and the little kindnesses we exhibit towards each other, that help to make this life pleasant and agreeable.

It is so in science; it is so in mechanism; and it is in dentistry. It is a little thing to say "keep your excavators and pluggers sharp, and see that they are in order, before you commence upon a tooth; but upon that depends the perfection and finish of your fillings. It is a little thing to say, "prepare well the corners and edges of your cavities; but upon that depends the durability and permanence of your plug." It is a little thing to say, "be sure that your fillings are kept entirely free from moisture; but upon that depends the thorough welding, or uniting the particles of gold. It is a little thing to say, "be patient, be thorough, be careful, be strong, and give every one who employs you, your *best gift*," but upon that alone depends your success as a dentist. It is a little thing to say, "be clean, be gentle, be modest, be manly;" but upon that depends your acceptability as a dentist. These things are, to the dentist, what the rains and the dews are to the plants and the flowers; they serve to make them flourish and grow, and become ornaments to society, and to the world. So it is in artificial dentures; the little things are the final adjustment or articulation, to see that every tooth impinges against every other tooth, so that mastication is perfect. Millstones will not grind, unless they touch each other, nor scissors cut well, unless the edges fit nicely together.

Much of the success of vulcanite work depends upon the quality of the plaster. Michigan plaster is the best I have ever used. Models made of Grand Rapids plaster are as hard as brass, (so to speak.) The most delicate points will be preserved through the whole process of vulcanizing; and no amount of steam or pressure will mar the surface, or render it unfit for use. It is a little thing to say to you, put your plaster into the water, rather then pour your water upon the plaster when mixing it: but by so doing you will have a more perfect impression or cast. It is hardly worth mentioning, to say, be careful not to put too much oil into your impression, before pouring your plaster; but oil occupies space, and the imperfections will be seen through the whole work, till it finally appears in a roughness upon the under side of the plate, when vulcanized.

This is the little atom of arsenic combined with creosote, that softens the pain in a raging tooth, when it is vehemently beating in its prison cell, almost enough to drive one to distraction, and quietly distroy its vitality, and renders it capable of being exterminated without pain or trouble.

It is the tiny blade of the lancet that depletes the vascular system, and restores harmony and peace, where all was discord and anguish, It is the little pellet of gold, or cotton, forced into the foramen of the tooth, that expels the death gas that causes suppuration and ulceration. It is the welding of each little particle of gold upon every other piece, that preserves the tooth, and restores its contour and beauty. Pluggers grow dull as well as excavators, and every one should possess a *knife-blade oil stone* to dress them, and keep them in order; and each pellet of gold should be prepared to receive the next pellet, and so on till the plug is completed; a good plug was never made with a dull instrument.

Gentlemen of the Convention, I hail, with satisfaction, the return of this anniversary of this Michigan State Convention, there is much to be proud of, it was in this convention that the first *Clinical operations* were introduced, that have since become so popular, and that have done so much to sustain and elevate the profession. You will forgive the earnestness and freedom with which I have spoken; but it is the voice of one who has, for thirty years, been a Dentist, and one who, for more than twenty, has worked with a single idea; and that has been the preservation of the natural teeth.

